# ﻿First and second instar larvae and adults of a new *Homidia* species (Collembola, Entomobryidae) recorded from Xizang Autonomous Region with three new records

**DOI:** 10.3897/zookeys.1089.73418

**Published:** 2022-03-16

**Authors:** Ling-bin Xiang, Shu-sheng Zhang, Lei-lei Liu, Zhi-xiang Pan

**Affiliations:** 1 School of Life Sciences, Taizhou University, Taizhou, Zhejiang 318000, China Taizhou University Taizhou China; 2 Wuyanling National Nature Reserve, Wenzhou, Zhejiang 325500, China Wuyanling National Nature Reserve Wenzhou China

**Keywords:** Entomobryini, key, larvae, taxonomy, Xizang

## Abstract

Three new recorded species of genus *Homidia* were collected from Xizang Autonomous Region, China, in the present paper. Among them, a new species, *Homidiabreviseta* Pan, **sp. nov.**, is included in the present paper. This new species can be identified by having a single uninterrupted dark band on central thoracic segment III; 14 macrochaetae on abdominal segment I and seven on the posterior central abdominal segment IV (half segment); and very short bothriotricha on abdominal segments II–IV. It can be easily discriminated from similar species of *Homidia* by its colour pattern, chaetotaxy of the labium, and abdominal segments I and IV. The chaetotaxy of the first and second instar larvae of this new species and a key to four species of genus *Homidia* from Xizang are also provided.

## ﻿Introduction

The genus *Homidia* Börner, 1906 is collembolan taxon widely distributed in southeast China and is generally found in every habitat, such as in leaf litter of forest, farmland, vegetable field, residential area and so on. This genus was established as a subgenus of *Entomobrya* (Rondani, 1861) by Börner (1906) and later raised to the generic level by Denis (1929). The significant character for the identification is that the dens bears spines and abdominal segment IV has an anterior series of macrochaetae transversely arranged as “eyebrows” in adults. Also, individuals with transverse bands, spots, or without pigment on the dorsal body are distinctive. *Homidia* species are good at jumping and large enough to be seen in wild by the naked eye. To date, 74 species of this genus have been reported worldwide (Bellinger et al. 1996–2021), and 42 are recorded from China (Ma and Pan 2017; Zhuo et al. 2018; Pan and Yang 2019; Pan and Ma 2021). However, among them only one species, *Homidiatibetensis* Chen & Zhong, 1998, was reported from Xizang.

Lhasa is the administrative centre of Xizang Autonomous Region, and with an altitude around 3600 m, it is one of the highest altitude cities in the world. Annual sunshine averages 3000 h and rainfall 200–510 mm. The climatic conditions results in unique biodiversity, including among Collembola. In order to gather more information about the diversity of Collembola from this region, we spent several days collecting around Lhasa in August 2019. Among the collected material, we found two new records and one new species of the genus *Homidia*. The chaetotaxy of the adult as well as the first and second instar larvae of the new species is described in detail. A comparison of the new species with the most similar species of the genus *Homidia* is provided. A checklist of all *Homidia* species found from Xizang is included as well as a key to separate them.

## ﻿Materials and methods

Collembolan individuals were sieved from leaf litter in the field, collected with an aspirator, and stored in 99% ethanol at –20 C in the laboratory. Specimens were photographed using a Nikon DS-Fi1 camera mounted onto a Nikon SMZ1000 stereomicroscope, then cleared in lactic acid, mounted in Hoyer’s medium under a coverslip, and examined with a Nikon 80i phase-contrast microscope. Lengths of morphological structures were measured from specimens in ethanol by NIS-Elements 3.1 software. Photographs, illustrations, and labels were enhanced by Photoshop CS5 (Abode Systems).

Dorsal chaetotaxy is provided for only one side of the body. The nomenclature of cephalic chaetotaxy, labial palp, labial chaetae, and dorsal thoracic and abdominal chaetotaxy follows the systems of Rueda and Jordana (2020), Fjellberg (1998), Gisin (1967), and Szeptycki (1979), respectively.

Specimens and all types are deposited in the School of Life Sciences, Taizhou University (**TZU**).

### ﻿Abbreviations

**Abd.** abdominal segment;

**Ant.** antennal segment;

**Gr.** group;

**mac** macrochaeta/e;

**mic** microchaeta/e;

**ms** specialized microchaeta/e;

**sens** specialized ordinary chaeta(e);

**S-chaeta/e** specialized chaeta/e, including ms and sens;

**Th. **thoracic segment;

**VT** ventral tube;

**l.p.** lateral process;

**asl** above sea level.

## ﻿Taxonomic account

**Figures 1–3.**
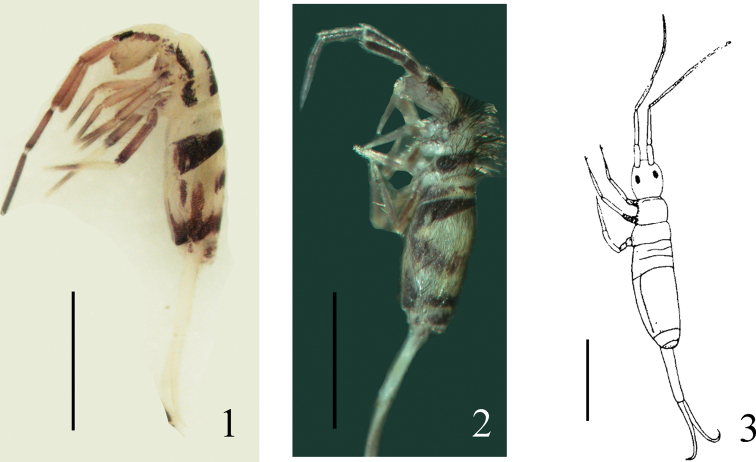
Colour pattern of *Homidia* species recorded from Xizang **1***Homidiasichuanensis***2***Homidiasinensis***3***Homidiatibetensis* (following Chen and Zhong 1998). Scale bars: 1000 μm

**Figures 4–9.**
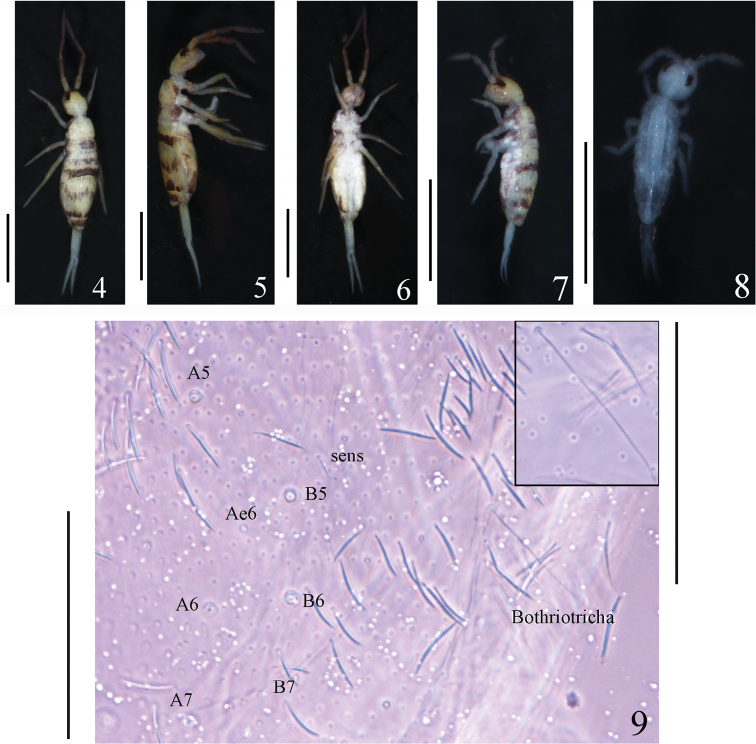
*Homidiabreviseta* Pan, sp. nov. **4–9** habitus **4** dorsal view of adults **5** lateral view of adults **6** ventral view of adults **7** dorsal view of subadults **8** dorsal view of the first instar larvae **9** dorsal view of Abd. IV, showing mac sockets and short bothriotricha. Scale bars: 1000 μm (**4–6**); 500 μm (**7–8**); 50 μm (**9**; left bar corresponds to large figure, right one to inset).

Fourteen samples (4687–4700) were collected in total from Lhasa from 1-VIII-2019 to 8-VIII-2019. The collection included two new records and one new species of the genus *Homidia: Homidiasichuanensis*Jia et al., 2010, *Homidiasinensis* Denis, 1929, *Homidiabreviseta* Pan, sp. nov. (Figs 1, 2, 4–8). A fourth species, *Homidiatibetensis* Chen & Zhong, 1998 (Fig. 3), which had been recorded from Xizang in a previous study (Chen and Zhong 1998), was absent from the present sampling.

The sampling information of three *Homidia* species recorded here are listed in Table 1. *Homidiasichuanensis* was described by Jia et al. (2010) from Sichuan Province, China, and is identified by its colour pattern and the presence of mac p4 and A6–A10 on Th. III and Abd. IV, respectively. It is widely distributed in western China, from Sichuan Province to Guangxi Zhuang Autonomous Region (recorded in our collection S09022603). *Homidiasinensis* was reported by Denis (1929) from Foochow, Fujiang Provnice, China, and is distinct from other species of *Homidia* by its colour pattern, chaetotaxy of the labium and Abd. I, III, and IV. It has a wide distribution, and we found it in most regions of China. *Homidiatibetensis* was described by Chen and Zhong (1998) from Xizang and is only know from there, but the detailed collecting information is not provided in the original description. This species, which is well-characterized morphologically by its colour pattern and chaetotaxy, is not included in our collections.

**Table 1.** Sampling information of *Homidia* species from Xizang Autonomous Region of China in the present study. All specimens were collected from Chengguan District of Lhasa City, in Xizang.

Sample no.	Location	Coordinates	asl (m)	Habitat	Collector	Species
4688	Lalu National Wetland Park	29°28'5.71"N, 91°4'55.15"E	3603±5	Leaf litter of white poplar forest	Z-X. Pan, C-C. Si	*H.sichuanensis*
4692	Gesan Flower Park	29°39'59.57"N, 91°7'18.38"E	3634±5	Leaf litter of family Rosaceae	Z-X. Pan, C-C. Si, J-F, Jia	*H.breviseta* sp. nov.
4696	Nanshan Park	29°38'15.69"N, 91°6'50.16"E	3633±5	Leaf litter of *Populussimonii*	Z-X. Pan, C-C. Si	*H.sichuanensis*
4698	Nongke Road, Germplasm Center of Xizang	29°38'27.28"N, 91°1'55.58"E	3584±5	Leaf litter of family Asteraceae	Z-X. Pan, J-F, Jia	*H.sinensis*

### ﻿Key to the *Homidia* species from Xizang

**Table:** 

1	Dorsal body with distinct transverse dark bands	**2**
–	Dorsal body without transverse dark band	***H.tibetensis***
2	Lateral head without longitudinal dark band	**3**
–	Lateral head with longitudinal dark bands	***H.sichuanensis***
3	Mac m3ei present on Abd. II and a3 absent on Abd. III	***H.breviseta* Pan, sp. nov.**
–	Mac m3ei absent on Abd. II and a3 present on Abd. III	***H.sinensis***

#### ﻿ Homidia breviseta

Taxon classificationAnimaliaEntomobryomorphaEntomobryidae

Pan sp. nov.

44FEE081-5AE4-5657-B318-970AC9EA6B6A

http://zoobank.org/F1734E97-767C-44B8-B096-218E146502B5

Figures 1–3 , 4–9 , 10–19 , 20–23 , 24–31 , 32–39 , 40–42 , 43–45 , 46–51

##### Type material.

***Holotype*.** 1♀ on slide, **China**, Xizang autonomous region, Lhasa city, Chengguan District, Gesan flower park, 29°39'59.5764"N, 91°7'18.3828"E, 3634±5 m asl, sample number 4692, collected by Z-X Pan, C-C Si, and F-H Jia, 3-VIII-2019. ***Paratypes*.** 7♀adults, 1 first and 1 second instar larva on slides and 5 adults in ethanol, same data as holotype.

##### Descriptions of adults.

***Size*.** Body length up to 1.62 mm. ***Colour pattern*.** Ground colour yellow-white in ethanol. Eye patches dark blue. Antennae gradually darker from Ant. I to Ant. IV. A dark narrow transverse band between basal antennae. Lateral Th. II–III with longitudinal bands, and dorsal Th. III with central transverse uninterrupted dark band. Coxa with dark pigment. Dorsal Abd. II and Abd. IV with central irregular dark bands. Dorsal Abd. III and Abd. V from anterior to posterior margin with dark transverse bands, and Abd. III with two lateral unpigmented areas. Dorsal Abd. IV with two middle and posterior transverse bands, the central one interrupted by a middle line (Figs 4, 5). Ventral side of body and VT pale white, without pigment (Fig. 6). Subadults with the same colour pattern as adults, but paler (Fig. 7).

**Figures 10–19.**
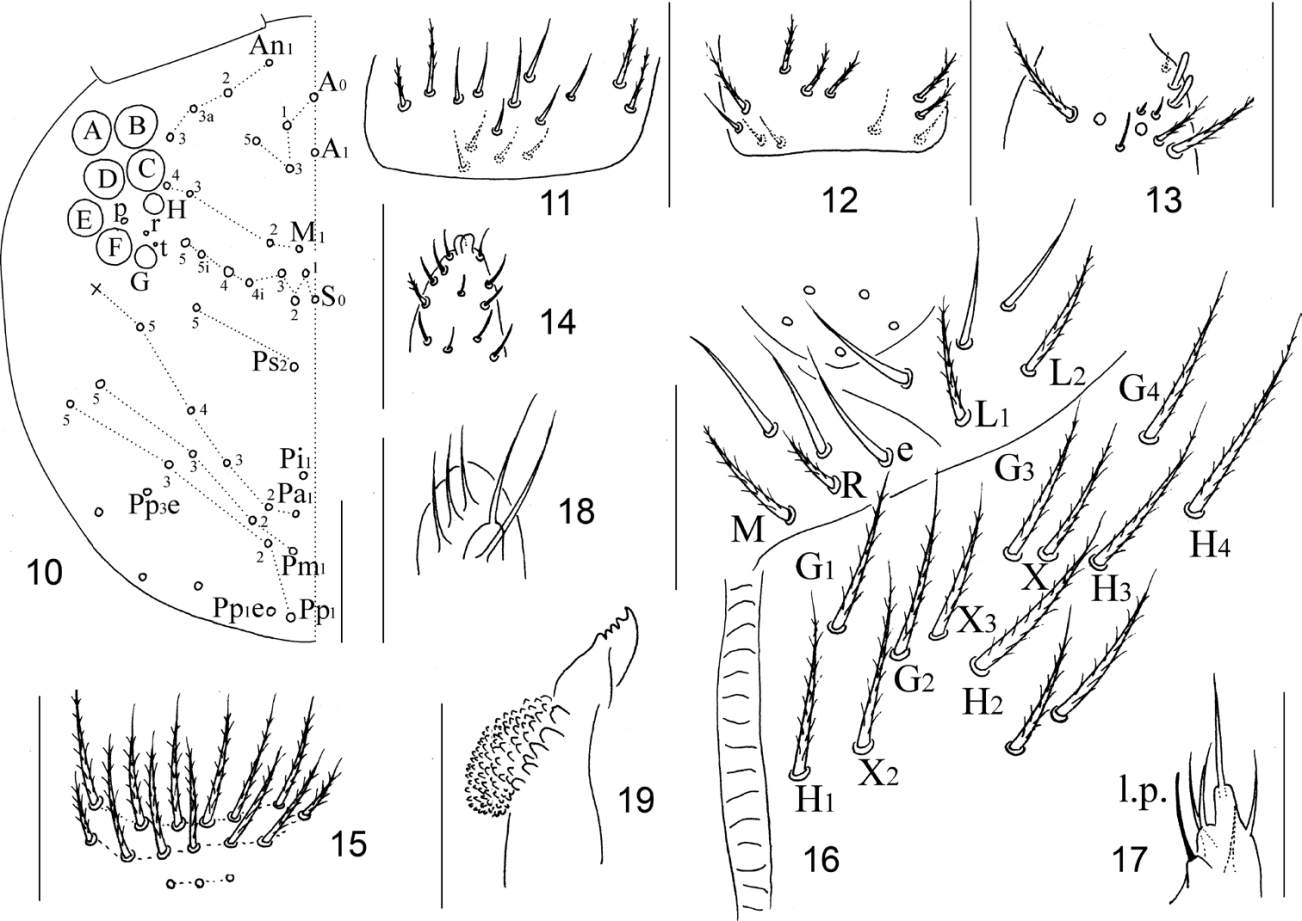
Adults of *Homidiabreviseta* Pan sp. nov. **10** cephalic chaetotaxy on dorsal side **11** base of Ant. I **12** base of Ant. II **13**Ant. III organ **14** distal part of Ant. IV **15** clypeal chaetotaxy **16** labium **17** labial papilla E **18** maxillary outer lobe **19** right mandible. Scale bars: 50 μm

***Head*.** Eyes 8+8, G and H smaller than others and always difficult to observe under light microscope; three chaetae (p, r, and t) within eye patches, with p largest (Fig. 10). Antenna 1.56–2.16 times as long as cephalic diagonal; antennal segments ratio as I:II:III:IV = 1:1.11–1.72: 1.15–1.76:1.97–2.73. Ant. I base with seven (rarely as three) dorsal smooth mic and four ventral (Fig. 11). Ant. II base with five smooth mic (Fig. 12). Ant. III organ with two rod-like and three short guard S-chaetae (Fig. 13). Apical bulb on Ant. IV bilobed (Fig. 14). Prelabral and labral chaetae as 4/5, 5, 4, all smooth; without labral papillae. Clypeus with 16 (6/7/3) chaetae in three lines (Fig. 15). Cephalic chaetotaxy on dorsal side shown in Fig. 10, An series with four (An1–3, An3a), A series with four (A0, A1, A3, A5), M series with four (M1–4), S series with eight (S0–5, S4i, S5i), P series with 18 (Ps2, Ps5, Pi1, Pa1–5, Pm1–3, Pm5, Pp1–3, Pp5, Pp1e, Pp3e) mac. Chaetae on labium basis as MReL_1_L_2_, chaeta e smooth; postlabial chaetae not expanded, with G_1–4_, H_1–4_, X_2_, X_3_, X all ciliate, X_4_ unclear; five proximal chaetae (Fig. 16). Five papillae A–E on labial palp with 0, 5, 0, 4, 3 guard chaetae, respectively; l.p. normal, with tip beyond apex of papilla E (Fig. 17). Maxillary outer lobe with single apical chaeta, one subapical chaeta, and three sublobal hairs on sublobal plate; subapical chaeta subequal in length to apical one (Fig. 18). Mandible with 4/5 apical teeth and basal strong molar plate (Fig. 19)

**Figures 20–23.**
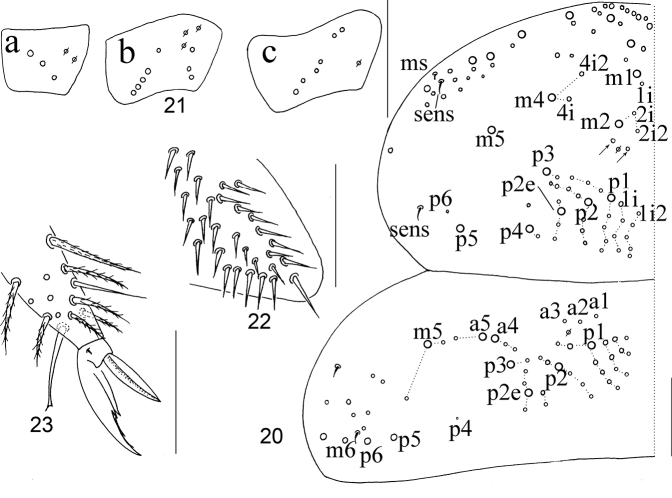
Adults of *Homidiabreviseta* Pan, sp. nov. **20** chaetotaxy of Th. II–III **21** coxae (**a** fore leg **b** mid leg **c** hind leg) **22** trochanteral organ **23** distal part of tibiotarsus and claw of hind leg. Scale bars: 50 μm.

***Thorax*.** Complete body sens from Th. II to Abd. IV as 2, 2/1, 2, 2, 28 (26 elongate and two of normal length), 3, ms as 1, 0/1, 0, 1, 0, 0. Th. II with seven medio-medial (m1, m1i, m2, m2i and m2i2 and other two additional mes; arrow shown in Fig. 20), three medio-sublateral (m4, m4i and m4i2) mac and three S-chaetae (ms antero-external to sens); posterior with 39–43 mac; p6 as mic. Th. III with 42–47 mac and two sens; p5, p6 and m6 as mac, p4 as mic (Fig. 20). Coxal macrochaetal formula as 3 (two pseudopores)/4+1, 3 (three pseudopores)/ 4+2 (one pseudopore) mac (Fig. 21). Trochanteral organ with 31–40 smooth chaetae, six or seven in ventral line, and five or six in posterior line (Fig. 22). Inner side of tibiotarsus with slightly ciliated chaetae. Tenent hairs clavate, slightly shorter than inner edge of unguis in length. Unguis with four inner and two lateral teeth. Unguiculus lanceolate with outer edge smooth (Fig. 23).

**Figures 24–31.**
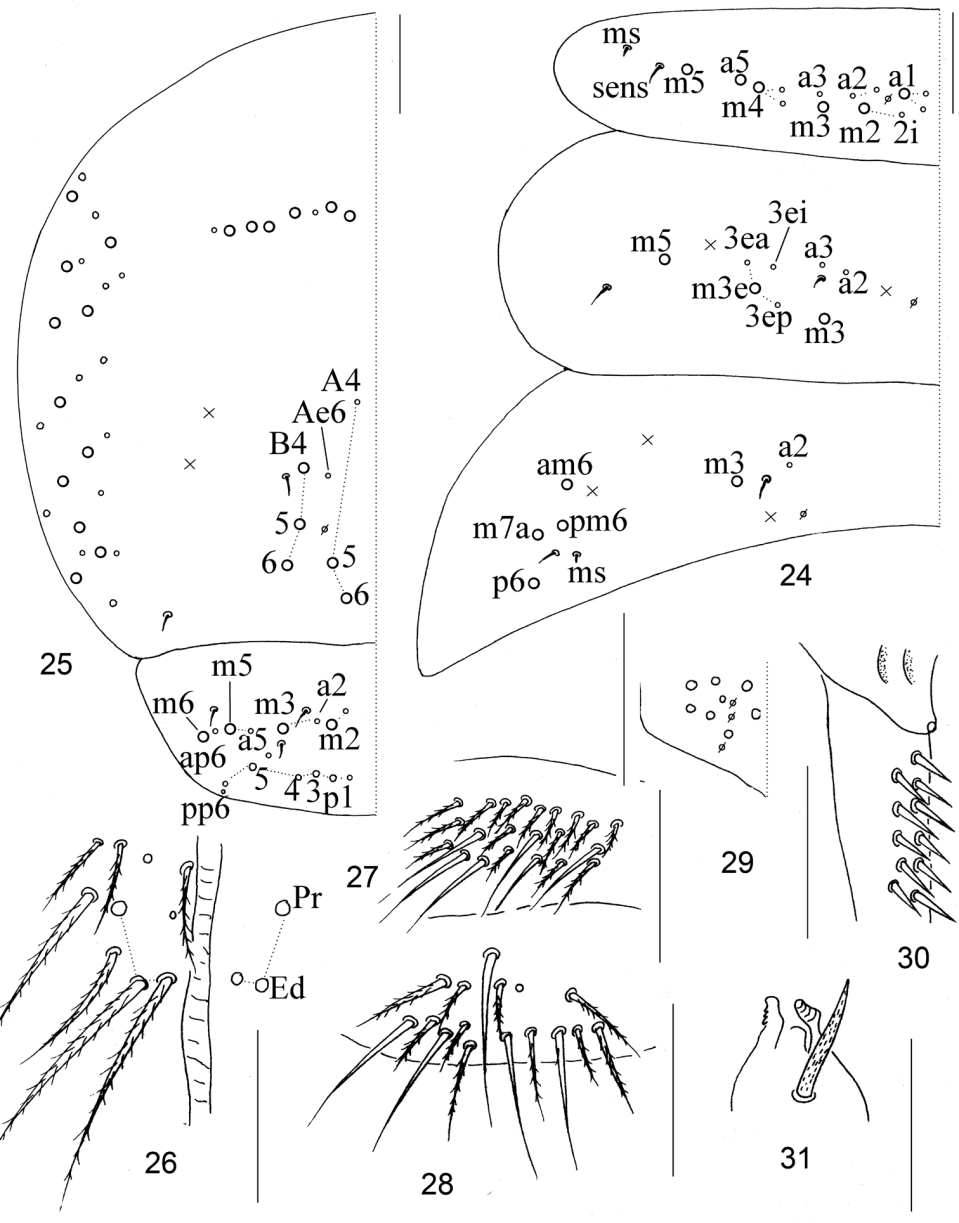
adults of *Homidiabreviseta* Pan, sp. nov. **24** chaetotaxy of Abd. I–III **25** chaetotaxy of Abd. IV–V **26** anterior face of VT**27** lateral flap of VT**28** posterior face of VT**29** manubrial plaque **30** basal part of dens **31** tenaculum. Scale bars: 50 μm.

***Abdomen*.**Abd. IV 5–8 times longer than Abd. III along the dorsal axis. Abd. I with 14 mac (a1–3, a5, a1a, a1i, a2i, m2–5, m2i, m4i, m4p) and two S-chaetae (ms antero-external to sens). Abd. II with seven central (a2, a3, m3, m3e, m3ea and m3ep, m3ei) and one lateral (m5) mac. Abd. III with two central (a2 and m3) and two lateral (am6, pm6, p6 and m7) mac, two sens and one ms (Fig. 24). Abd. IV with 26 elongated and two normal length sens, and 6–9 mac arranged in anterior transversal line; postero-central area with seven mac (A4–6, B4–6, Ae6; one individual examined with Ae4 and Ae7), bothriotricha short and no more than two times as long as normal ciliate chaetae (Figs 9, 25). Abd. V with three sens, the middle one posterior to m3, the lateral one between chaetae a5 and m5; a1 as mic; a3, m3, m5, a5, m5, and a6 as mac (Fig. 25). Anterior face of VT with many ciliate chaetae, 3+3 of them as mac, the line connecting proximal (Pr) and external-distal (Ed) mac obliquely to median furrow (Fig. 26); lateral flap with 5–7 smooth and 10–17 ciliate chaetae on each side (Fig. 27); apical posterior face as five (2+1+2) smooth chaetae (two specimens examined here with four smooth chaetae) (Fig. 28). Manubrial plate with three pseudopores and eight or nine ciliate chaetae (Fig. 29). Dens with 23–33 inner spines, distal smooth part slightly shorter than mucro (only basal part shown in Fig. 30). Mucro bidentate with subapical tooth larger than apical one; basal spine short, with tip reaching subapical tooth. Tenaculum with 4+4 teeth and single large, multi-laterally, basally ciliate chaeta (Fig. 31).

##### Description of the first instar larva.

***Size*.** Body length up to 0.59 mm. ***Colour pattern*.** Ground colour whitish, only eye patches dark blue, others all without pigment (Fig. 8).

***Body*.** Complete tergal sens from Th. II to Abd. V as 2, 2/1, 2, 2, 28, 3, ms as 1, 0/1, 0, 1, 0, 0. Cephalic chaetotaxy on dorsal side with three (An1–3), six (A0–5), four (M1–4), six (S0–5) mac of An, A, M, S series, respectively; eyes 8+8, eye patches with three chaetae (p, r, and t; p largest). Labium with three proximal chaetae, four chaetae (M, e, A and B) in basomedial field and five chaetae (C, D, F, L_1_ and L_2_) in basolateral field, chaetae M, L_1_ and L_2_ ciliate, and others smooth; posterior area of labium with two ciliate mac along median furrow (Fig. 33). Th. II with seven anterior (a1–7), six median (m1–2, m4–7), and six posterior (p1–6) primary chaetae arranged in three rows; chaetae a7, m2, m5, m7, and p4, p6 as mic, others as mac, and with three S-chaetae (ms antero-external to sens). Th. III with seven anterior (a1–7), five median (m1, m4–7), and six posterior (p1–6) primary chaetae arranged in three rows and two S-chaetae; chaetae a4, a7, m1, m4, m5, m7, and p4–6 as mic, others as mac. Abd. I with five anterior (a1–3, a5–6), five median (m2–6), and two posterior (p5–6) primary chaetae arranged in three rows and two S-chaetae (ms antero-external to sens); chaetae m2–m4 as mac, others as mic. Abd. II with six anterior (a1–3, a5–7), six median (m2–7), and four posterior (p4–7) primary chaetae arranged in three rows, an additional chaeta external to p7 and two S-chaetae; chaetae m3 and m5 as mac, a5 and m2 as bothriotricha, others as mic. Abd. III with six anterior (a1–3, a5–7), seven median (m2–5, am6, pm6, m7), and four posterior (p4–7) primary chaetae arranged in three rows, five additional chaetae in lateral region, and three S-chaetae (one ms and two sens); chaeta m3 as mac, m2, a5, and m5 as bothriotricha, others as mic (Fig. 34). Abd. IV with five (A1–4, A6), six (B1–6), four (C1–4), seven (T1–7), three (D1–3), three (E1–3), and three (F1–3) primary ciliate chaetae arranged in seven longitudinal lines, one side with an additional ciliate chaeta between C2 and C3 (shown by arrow in Fig. 35), and 26 elongated and two normal sens; T2 and T4 as bothriotricha. Abd. V with 13 primary chaetae (m2, m3 and m5 as mac; others as mic) and three sens, the median sens posterior to m3 (Fig. 35).

**Figures 32–39.**
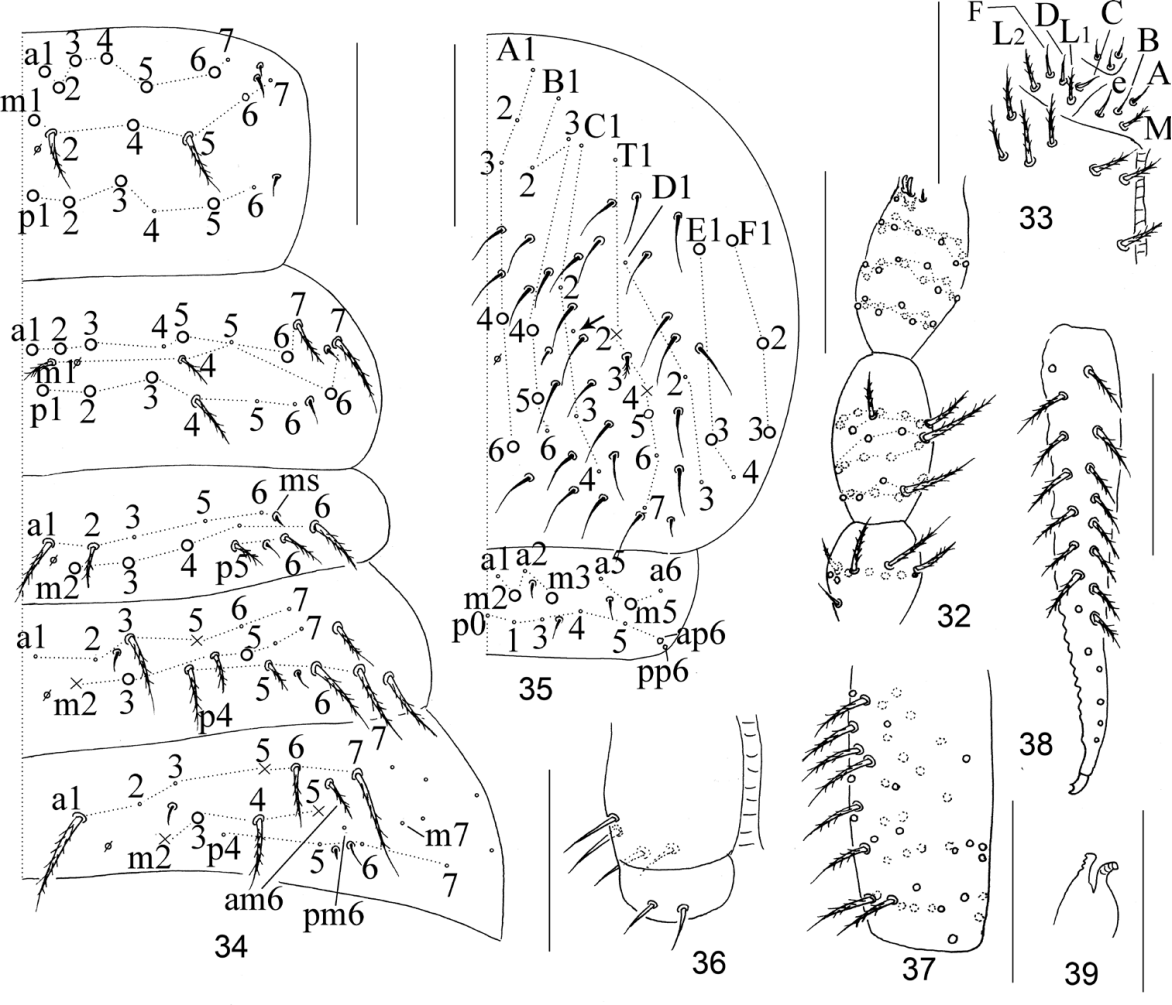
The first instar larva of *Homidiabreviseta* Pan, sp. nov. **32** chaetotaxy of Ant. I–III **33** labium **34** chaetotaxy of Th. II–Abd. III **35** chaetotaxy of Abd. IV–V **36** ventral tube **37** manubrium **38** dens **39** tenaculum. Scale bars: 50 μm.

**Figures 40–42.**
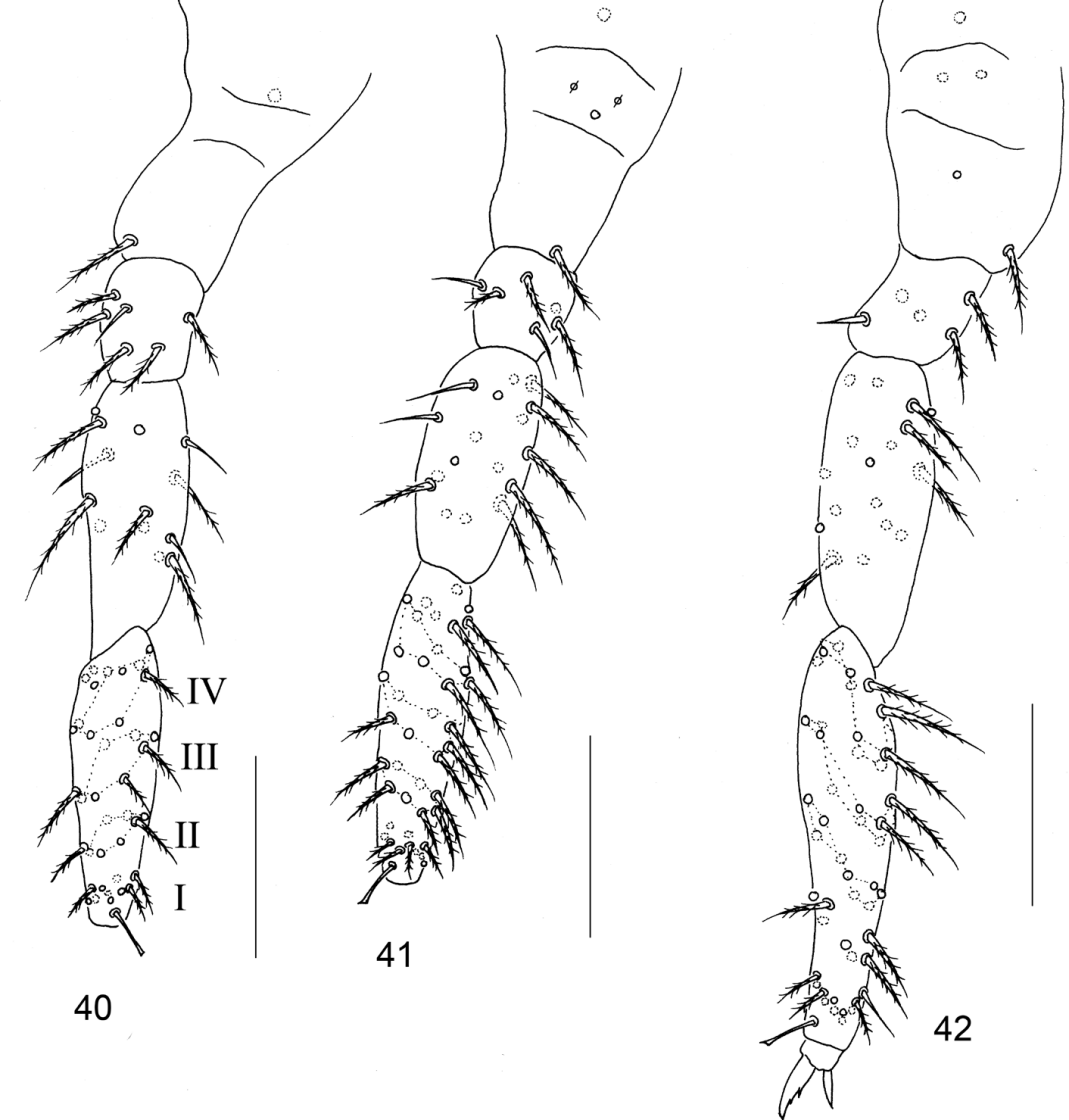
Left legs of the first instar larva of *Homidiabreviseta* Pan, sp. nov. **40** fore leg **41** mid leg **42** hind leg. Scale bars: 50 μm.

***Appendages*.**Ant. I with 11 ciliate chaetae arranged in one whole and one basal smooth chaeta. Ant. II with 25 ciliate chaetae, arranged in three wholes (from basis to apex as 8/8/9), basis without smooth spiny chaetae. Ant. III with 37 ciliate chaetae arranged in four wholes (11/12/13/2) and five S-chaetae (Ant. III organ) (Fig. 32). Primary chaetae on Ant. IV unclear. Ventral tube with two smooth chaetae on the posterior face and on each lateral flap, anterior face without chaetae (Fig. 36). Manubrium with 46 ciliate chaetae (Fig. 37); dens with numerous ciliate chaetae, without inner dental spines; chaetae bs2, bs1, pi unclear; mucro with subapical tooth larger than apical one, basal spine absent (Fig. 38). Tenaculum with 4+4 teeth and without basal chaetae (Fig. 39). Four segments of fore, mid and hind leg with numerous chaetae, subcoxae with 1, 2, 3 ciliate chaetae, coxae with 1, 1, 2 ciliate chaetae, pseudopore(s) unclear; trochanters with six (one smooth), six (two smooth), five (one smooth and one spine like) chaetae; femurs with 17 (three smooth), 17 (smooth chaetae unclear), 17 (two smooth) chaetae; tibiotarsus with 39 (10/8/8/8/4 ciliate and one tenent hair), 41 (10/8/8/8/6 ciliate and one tenent hair), 48 (10/7/9/9/9/2, one tenent hair and one inner smooth chaetae) ciliate chaetae (Figs 40–42).

##### Description of the second instar larva.

***Colour pattern*.** Ground colour whitish; eye patches dark blue. The colour pattern of the second instar larva is similar to adult, but slighter.

The chaetotaxy of the second instar larva is more complex than first instar, and several primary chaetae with secondary chaetae present in the second instar (Figs 43–54). The detailed comparison between these two instars are tabulated in Table 3.

**Table 3.** Detailed comparison of chaetotaxy between the first and second instar larvae of *Homidiabreviseta* sp. nov.

Characters			First instar	Second instar
Th. II	a/m/p series		7/6/6	11/9/11
Th. III	a/m/p series		7/5/6	8/6/9
Abd. I	a/m/p series		5/5/2	5/6/3
Abd. II	a/m/p series		6/6/4	6/9 (m3e present) /5
Abd. III	a/m/p series		6/6/4	8/?/4
Abd. IV	ciliate chaetae		30	53
Abd. V	ciliate chaetae		13	27 (m3a present)
Fore leg	subcoxa		1	2
	coxa		1	2
	trochanters		6 (4c*+2s^†^)	6 (4c+2s)
	femurs		17 (14c+3s)	18
	tibiotarsus^3^	whole I	10	10
		whole II	8	8
		whole III	8	8
		whole IV	8	8
		additional	4	7
Mid leg	subcoxa		2	5
	coxa		1	5
	trochanters		6 (4c+2s)	7
	femurs		17	21 (19c+2s)
	tibiotarsus^3^	whole I	10	10
		whole II	8	8
		whole III	8	8
		whole IV	8	8
		additional	6	3
Hind leg	subcoxa		3	2
	coxa		2	5
	trochanters		5 (1 spine)	6 (1 spine)
	femurs		17 (15c+2s)	22 (21c+1s)
Hind leg	tibiotarsus^3^	whole I	10	10
		whole II	7	9
		whole III	9	9
		whole IV	9	9
		whole V	9+2	9
Labium	proximal chaetae		3s	5s
Ventral tube	anterior face		0	1c+1c
	lateral flap		2s+2s	5s+5s
	posterior face		2s	3s
Tenaculum	basal chaeta		0	1
Manubrium	chaetae		46c	77c
Mucro	basal spine		absent	present

*Ciliate, ^†^Smooth, ‡Chaetae on tibiotarsus, not including tenent hair and smooth apical chaeta.

**Figures 43–45.**
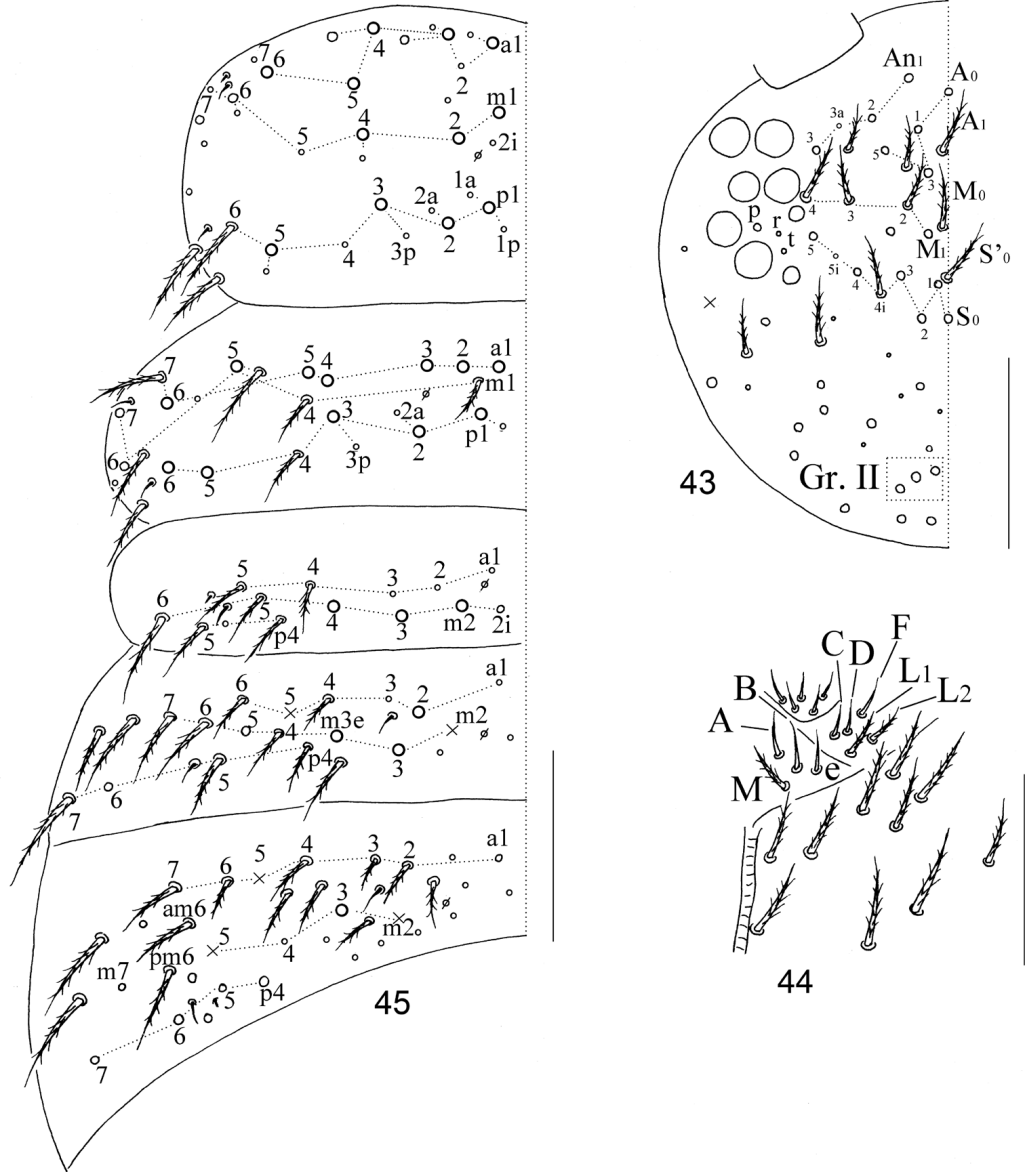
The second instar larva of *Homidiabreviseta* Pan, sp. nov. **43** cephalic chaetotaxy **44** labium **45** chaetotaxy of Th. II–Abd. III. Scale bars: 50 μm.

##### Ecology.

All stages were found in leaf litter of the Family Rosaceae.

**Figures 46–51.**
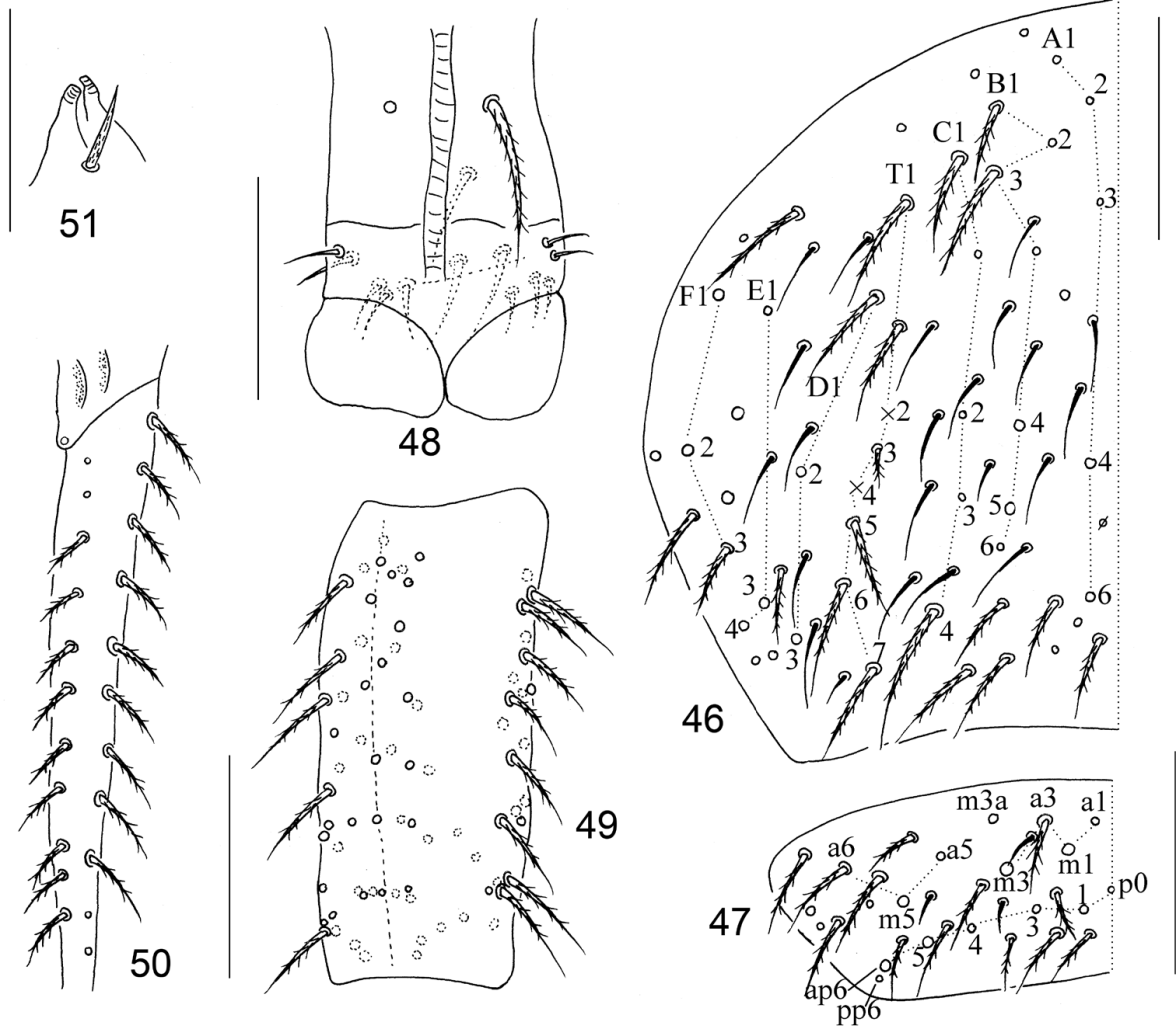
The second instar larva of *Homidiabreviseta* Pan, sp. nov. **46** chaetotaxy of Abd. IV **47** chaetotaxy of Abd. V **48** ventral tube **49** manubrium **50** dens **51** tenaculum. Scale bars: 50 μm.

##### Etymology.

The specific epithet refers to the very short chaeta bothriotricha on dorsal Abd. II–IV (*brevi* and *seta*).

**Figures 52–54.**
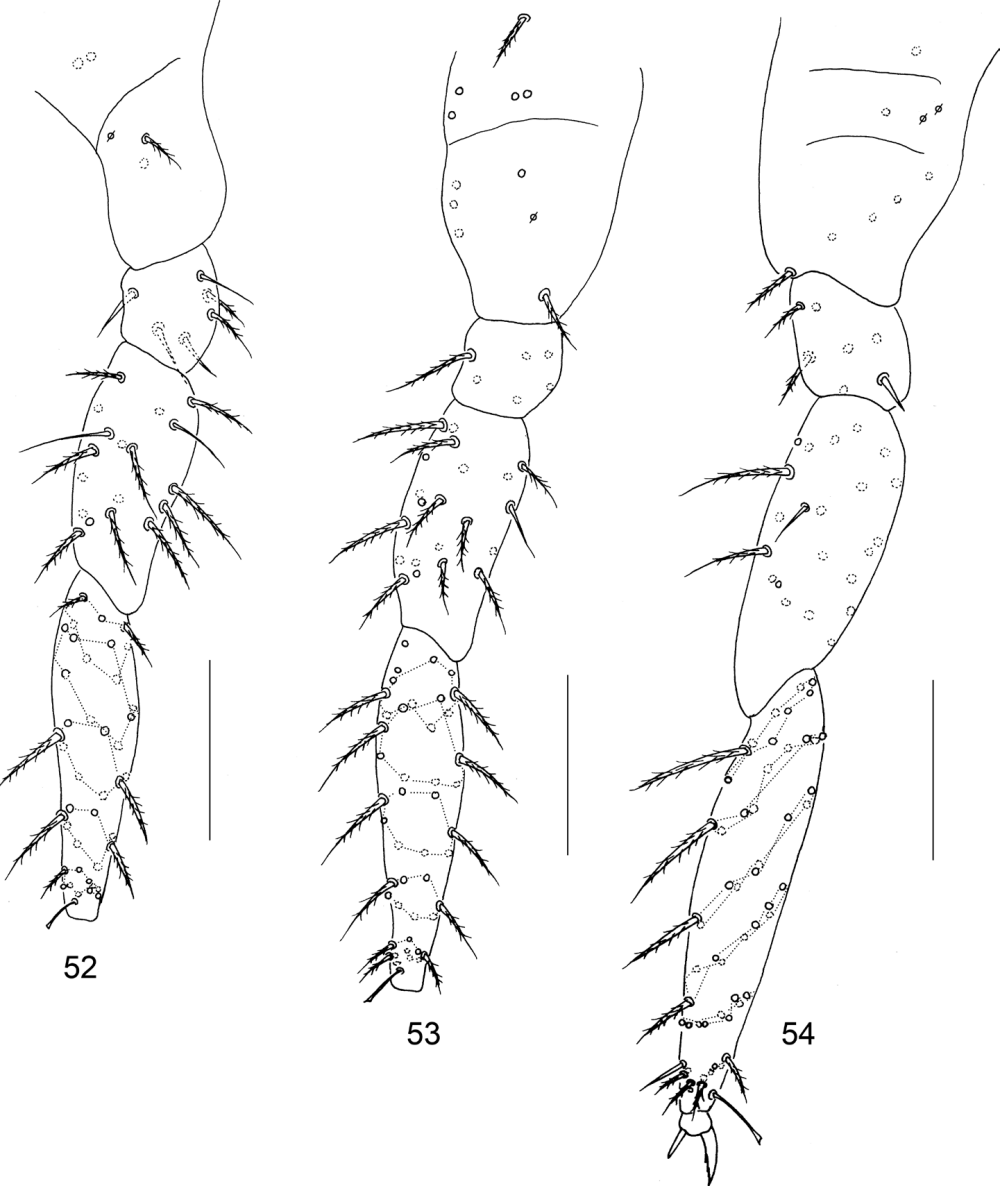
Legs of the second instar larva of *Homidiabreviseta* Pan, sp. nov. **52** fore leg **53** mid leg **54** hind leg. Scale bars: 50 μm.

##### Remarks.

This new species is mostly similar to *Homidiasimilis* Szeptycki, 1973 in having Th. III, Abd. III, and the middle and posterior of Abd. IV and Abd. V with transverse bands; in the chaetotaxy of the labium, head, Th. III, and Abd. II–III; and smooth chaetae on posterior face (five) and lateral flap (six) of VT. However, the new species can be differentiated from *H.similis* by the uninterrupted band on Th. III (interrupted by a central line in latter), a broad band from the anterior to posterior margin of Abd. III (anterior margin not pigmented in the latter), 14 mac on Abd. I (nine in the latter), and seven posterior central mac on Abd. IV (eight in the latter). Also, the new species is similar to *H.bilineata* Lee & Park, 1984 and *Homidiahuashanensis*Jia et al., 2005 in having mac on the dorsal head, Th. II–III, and Abd. II and chaetal formula on labium; however, they can be discriminated by colour pattern, chaetotaxy on Abd. I and posterior central Abd. IV. A detailed comparison of these four similar species is given in Table 2.

**Table 2.** Comparison between the new species and other similar species of *Homidia*.

Characters	*H.breviseta* sp. nov.	*H.similis*	*H.bilineata*	*H.huashanensis*
Pigment on central Th III	as one complete band	separated to two parts by middle line^*†^	without	not as band
Pigment on Abd. III reaching anterior margin	yes	no^*†^	no	no
Labial papilla	0	4 smooth^*†^	4 smooth^†^	0
Additional mac present posterior to m2 on central Th. II	2	0	not mentioned	0
Mac on coxa of hind leg	4+2	4+3*	4+1	4+2
Mac on Abd. I	14	9*	not mentioned	11-12 (mostly 12)
Mac between A and B series on posterior region of Abd. IV	1 (Ae6)	2 (Ae6 and Ae7)^†^	0	2 (Ae5 and Ae6) or 3 (Ae5–7)
Line connecting Pr to Ed on anterior face of VT to median furrow	oblique	parallel*	parallel	oblique
Distribution	Xizang Autonomous Region	Fujian and Zhejiang Province^‡^; Korea^*†^	Korea	Shaanxi Province

*Refers to the description by Szeptycki (1973), ^†^Refers to the description by Jordana (2012), ^‡^Refers to the description by Chen et al. (2011).

This species is the second species of genus *Homidia* described from Xizang, and it can be easily distinguished from the first new species recorded from this region (*H.tibetensis* Chen & Zhong, 1998) by the colour pattern (dorsal central pigments on Th. III and Abd. III in the new species, absent in *H.tibetensis*), chaetotaxy on the labial triangle (M2 absent in the new species, but present in *H.tibetensis*), Abd. I (14 mac in the new species, and 11 in *H.tibetensis*), and posterior part of Abd. IV (seven in new species and only two in *H.tibetensis*).

## Supplementary Material

XML Treatment for Homidia breviseta
